# Reply: Challenging the TAVR-Centric Approach in Asymptomatic Severe AS

**DOI:** 10.1016/j.jacadv.2025.102518

**Published:** 2026-01-17

**Authors:** Victor Dayan, Deepak L. Bhatt

**Affiliations:** a Centro Cardiovascular Universitario, Universidad de la Republica, Montevideo, Uruguay; b Mount Sinai Fuster Heart Hospital, Icahn School of Medicine at Mount Sinai, New York, New York, USA

We thank Dr Rose and colleagues for their thoughtful comments regarding our review on early intervention vs conservative management in asymptomatic severe aortic stenosis (AS).^1^ Their letter raises important considerations about procedural modality, surgical benchmarks, and patient selection that indeed merit ongoing scientific discussion. At the same time, these points underscore a broader challenge: current recommendations in asymptomatic AS must be grounded in randomized clinical trials with robust external validity and not extrapolated from procedural preferences or single-center experiences.

As highlighted by Dr Rose and colleagues, the RECOVERY (Randomized Comparison of Early Surgery vs Conventional Treatment in Very Severe Aortic Stenosis) trial reported a substantial mortality reduction with early surgical aortic valve replacement (SAVR). However, interpreting RECOVERY as definitive evidence for a surgical advantage in younger patients requires caution. RECOVERY enrolled only 145 patients at a single center in Korea, with exceptionally low operative risk profiles and intensive follow-up not easily reproduced in real-world practice.^2^ Similarly, the AVATAR (Aortic Valve Replacement Vs Conservative Treatment in Asymptomatic Severe Aortic Stenosis) trial—although important—was underpowered, prematurely stopped, and largely driven by a single enrolling institution.^3^ These characteristics inherently limit external validity and restrict generalizability to the broad and heterogeneous population of asymptomatic AS seen worldwide.

Conversely, the EARLY TAVR (Evaluation of TAVR Compared to Surveillance for Patients with Asymptomatic Severe Aortic Stenosis) trial, the largest randomized trial to date, enrolled 901 patients across multiple countries, providing a more contemporary reflection of practice variability and patient diversity.^4^ The mean age of included patients was 76 years which makes any comparison with RECOVERY challenging. Due to the wide age difference between transcatheter aortic valve replacement (TAVR) and SAVR trials, any attempt to compare techniques indirectly is prone to bias.

We agree with the correspondents that minimally invasive SAVR, sutureless prostheses, and rapid-deployment platforms are valuable advances. Yet these techniques, although promising, have never been evaluated in randomized trials against surveillance in asymptomatic patients, nor are they uniformly available across centers—further limiting generalizability. Likewise, concerns about TAVR durability, conduction disturbances, and paravalvular leak are valid, but only long-term randomized data can determine their clinical impact, particularly in younger patients.

As we emphasized in our review, recommendations for asymptomatic AS should not hinge on assumptions about procedural superiority—surgical or transcatheter—or on historical comparisons between contemporary TAVR and past eras of SAVR. Instead, they must arise from randomized evidence that includes diverse patient populations, standardized surveillance protocols, and outcomes meaningful across health care systems.

We concur that future studies should incorporate patient-reported outcomes, functional capacity, return-to-work metrics, and procedural access strategies as long as the follow-up is longer than 2 years. These dimensions are central to shared decision-making but are notably absent from current randomized data.

In summary, although procedural innovation is welcome and ongoing refinement of SAVR and TAVR is expected, clinical recommendations must be anchored in randomized trials with strong external validity, rather than extrapolated from technique-specific arguments. Until such evidence is available, a tailored approach—balancing procedural risk, surveillance feasibility, and patient-centered priorities—remains the most evidence-based strategy for asymptomatic severe AS.
